# Adolescent mentalizing and childhood emotional abuse: implications for depression, anxiety, and borderline personality disorder features

**DOI:** 10.3389/fpsyg.2023.1237735

**Published:** 2023-07-19

**Authors:** Gabriel Martin-Gagnon, Lina Normandin, Peter Fonagy, Karin Ensink

**Affiliations:** ^1^École de Psychologie, Université Laval, Québec, QC, Canada; ^2^Psychoanalysis Unit, Research Department of Clinical, Educational and Health Psychology, University College London, London, United Kingdom

**Keywords:** mentalizing, adolescent, borderline personality disorder, depression, anxiety

## Abstract

**Background:**

There is preliminary evidence that childhood emotional abuse (CEA) is a risk factor for adolescent mentalizing difficulties (Uncertainty/Confusion about mental states) and borderline personality features and that Uncertainty/Confusion about mental states mediate the relationship between CEA and adolescent borderline personality features, but these findings need replication. Furthermore, no previous studies have examined the relationship between adolescent mentalizing deficits, anxiety, and depression in the context of CEA.

**Objectives:**

This study examined the associations between CEA, adolescent borderline personality features, depression and anxiety symptoms and tested a pathway model where Uncertainty/Confusion about mental states mediates the relationships between CEA and adolescent borderline personality features, depression and anxiety symptoms.

**Method:**

A clinical sample of 94 adolescents completed the Reflective Function Questionnaire for Youth (RFQY) to assess mentalizing, the Childhood Experiences of Care and Abuse Questionnaire (CECA-Q), the Borderline Personality Disorders Features Scale (BPFS-C), and the Beck Youth Inventories for Depression (BDI-Y) and Anxiety (BAI-Y).

**Results:**

Uncertainty/Confusion about mental states partially mediated the relationship between CEA and borderline traits as well as anxiety. In addition, there was an indirect effect where CEA predicted Uncertainty/Confusion about mental states, which then predicted depression.

**Discussion:**

The findings are consistent with the mentalizing model of psychopathology and provide new evidence that Uncertainty/Confusion about mental states might be a critical mentalizing deficit that characterizes the associations between CEA and adolescent BPD features and depression and anxiety symptoms. Uncertainty/Confusion may be a transdiagnostic risk factor for adolescent psychological distress and dysfunction. We discuss the clinical implications.

## Introduction

Childhood Emotional Abuse (CEA) affects up to 48% of the general population (Goldsmith et al., 2013) and refers to recurrent invalidation, humiliation, criticism, and rejection on the part of caregivers (Hart and Brassard, 1991). CEA is a known risk factor for adolescent borderline personality disorder (BPD; Bounoua et al., 2015; Duval et al., 2018b; Porter et al., 2020), depression (Cohen et al., 2019; Humphreys et al., 2020), and anxiety (Dye, 2020; Guo et al., 2021). However, the processes through which CEA increases the risk of psychopathology in adolescents remain unclear (Fritz et al., 2018). Recent research suggests that CEA may induce mentalizing difficulties characterized by Uncertainty/Confusion about mental states, mediating the relationship between CEA and adolescent Borderline personality features (Duval et al., 2018b). Although mentalizing deficits are known as transdiagnostic risk factors for psychopathology (Fonagy et al., 2016; Luyten et al., 2020), further research is required to clarify how Uncertainty/Confusion about mental states relates to CEA and adolescent psychological difficulties such as depression and anxiety. In order to enhance interventions for adolescents in the context of CEA, it is crucial to elucidate the role of Uncertainty/Confusion in various psychological difficulties.

This study addresses these gaps in the literature by examining pathways involving CEA, mentalizing difficulties including Uncertainty/Confusion, and psychological difficulties, including BPD features, depression and anxiety in adolescent mental health service users.

### Development of mentalizing

Mentalizing (MZ), operationalized for research purposes as Reflective Functioning, is the uniquely human capacity involved in imagining and understanding others as well as ourselves in terms of intentional mental states such as feelings, wishes, and attitudes (Fonagy et al., 2002). MZ is a developmental achievement (Ensink and Mayes, 2010; Luyten and Fonagy, 2015). MZ develops optimally in secure attachment relationships where children feel safe sharing their difficulties, thoughts and feelings with a benign adult who can help them understand what they are experiencing (Fonagy et al., 2002; Ensink et al., 2017). On the other hand, childhood trauma negatively impacts the development of mentalizing (Ensink et al., 2014, 2015; Duval et al., 2018b; Huang et al., 2020). However, evidence shows that mentalizing can also be a resilience factor in the context of childhood maltreatment (Ensink et al., 2017; Berthelot et al., 2019; Borelli et al., 2021).

During adolescence, MZ undergoes a major reorganization which ultimately leads to the emergence of full mentalizing capacities in young adulthood (Blakemore, 2008; Taylor et al., 2013). Furthermore, MZ appears particularly important for engaging successfully with the biopsychosocial challenges of adolescence and is associated with better emotional regulation, socioemotional development, and identity consolidation (Braehler and Schwannauer, 2012). However, research suggests that adolescents with MZ impairments are more vulnerable to developing psychopathology (Hauser et al., 2006, Sharp et al., 2009; Duval et al., 2018a,b).

Frequently researched mentalizing impairments include Uncertainty/Confusion about mental states, hypermentalizing and hypomentalizing (Fonagy et al., 2002; Duval et al., 2018a). Uncertainty/Confusion about mental states refers to difficulties identifying and linking mental states, resulting in a state of confusion (Duval et al., 2018a). Typical examples of Uncertainty/Confusion include being puzzled by one’s mental state or that of others, or being unable to link intentionality with behavior (Duval et al., 2018a,b). On the other hand, Hypermentalizing, also known as pseudo-mentalizing or excessive certainty, has been defined as an over attribution of mental states without sufficient evidence (Sharp et al., 2013, 2016). Finally, hypomentalizing or low MZ is a reduced capacity to use MZ that prevents individuals from understanding/considering complex mental states (Fonagy et al., 2016).

### Mentalizing, BPD features, depression, and anxiety

Adolescent BPD is associated with general MZ deficits (Beck et al., 2017; Bo and Kongerslev, 2017; Norup and Bo, 2019), as well as specific MZ impairments such as Uncertainty/Confusion about mental states (Duval et al., 2018b), hypomentalizing (Vahidi et al., 2021) and hypermentalizing (Sharp et al., 2013, 2016; Somma et al., 2019; Penner et al., 2020). Duval et al. (2018a) found a much stronger relationship between BPD features and Uncertainty/Confusion about mental states (*r* = 0.76) compared with a weak to moderate strength relationship between BPD features and hypermentalizing measured with the MASC (*r* = 0.21). The strong correlations between Uncertainty/Confusion and BPD features obtained by Duval et al. (2018a) suggest that Uncertainty/Confusion about mental states may be a MZ difficulty characteristic of BPD, but further research is needed to examine this.

Regarding depression and MZ, research to date has primarily focused on adults and children (Ensink et al., 2017; Fischer-Kern and Tmej, 2019). Indeed, a recent meta-analysis concluded that in adults, depression severity is associated with the severity of MZ impairment (Fischer-Kern and Tmej, 2019). In addition, Li et al. (2020) found that adult depressive symptoms were positively associated with hypo and hypermentalizing. Regarding depression in youth, studies using the Child Attachment Interview rated for Reflective Functioning (Ensink et al., 2015) have shown that MZ about others mediated the relationship between sexual abuse and depression symptoms (Ensink et al., 2017). Associations between self-reported general MZ difficulties and adolescent depression are also evident in clinical samples (Belvederi Murri et al., 2016). Furthermore, internalizing symptoms are associated with hypo and hypermentalizing (Seyed Mousavi et al., 2021) and Uncertainty/Confusion about mental states (Duval et al., 2018a).

Research on anxiety and MZ is scarce, and the findings are divergent. Some studies report positive associations between anxiety and MZ deficits in adolescents (Ballespi et al., 2018; Seyed Mousavi et al., 2021), as well as social anxiety and hypermentalizing in young adults (Washburn et al., 2016; Ballespí et al., 2019). On the other hand, others have found that higher anxiety is associated with better MZ about self and attachment figures (Chow et al., 2017; Chevalier et al., 2021). Moreover, a recent meta-analysis of 105 studies concluded that anxious individuals might have modest general MZ impairments (Chevalier et al., 2023). However, the nature of the MZ deficits associated with anxiety symptoms remains unclear.

### Maltreatment, mentalizing, and psychopathology

According to the MZ developmental model of BPD, early maltreatment disrupts the development of MZ, leading to poorer affect regulation, self-other differentiation, and a low threshold for controlled MZ deactivation, which all contribute to the risk of developing BPD symptoms (Fonagy and Luyten, 2009; Duval et al., 2018b). Similar MZ developmental models have been proposed to explain the relationship between maltreatment, MZ, depression (Ensink et al., 2017; Luyten et al., 2019) and anxiety (Nolte et al., 2011).

There is empirical evidence of the MZ developmental model showing pathways from maltreatment through MZ to psychological difficulties in adults (Chiesa and Fonagy, 2014; Brüne et al., 2016; Ensink et al., 2020; Li et al., 2020), children (Ensink et al., 2017), and adolescents (Quek et al., 2017; Duval et al., 2018b). In adolescents, MZ mediated the relationship between CEA and adolescent BPD features (Quek et al., 2017; Duval et al., 2018b) and between childhood trauma and depression (Belvederi Murri et al., 2016). These previous findings are broadly consistent with the MZ developmental model of psychopathology but require replication. Furthermore, most of these studies do not specify the type of MZ impairment through which CEA impacts psychopathology, and further research is necessary to clarify the nature of the MZ difficulties associated with CEA and psychopathology. Moreover, to our knowledge, no previous studies have examined the mediating role of MZ in the relationship between CEA and anxiety in adolescents.

Recent evidence suggests that CEA may disrupt MZ and induce Uncertainty/Confusion about mental states (Duval et al., 2018b). Indeed, by repeatedly misattributing negative qualities and intentions to children, we would expect CEA to induce Uncertainty/Confusion in the child about their intentions, as well as confusion about the parents’ minds and intentions. Furthermore, by creating an environment that discourages a coherent discourse about mental states, CEA could undermine the development of a robust sense of self and the ability to interpret the intentions of others, ultimately resulting in psychopathology (Luyten and Fonagy, 2018). Further studies are needed to confirm these claims.

There is evidence that Uncertainty/Confusion about mental states contributes to psychological difficulties in youth (Duval et al., 2018a,b). Duval et al. (2018b) investigated the relationship between CEA, MZ, and BPD features in 263 adolescents from a community sample (aged 12–21). They found that Uncertainty/Confusion about mental states partially mediated the relationship between CEA and BPD features. To our knowledge, the study of Duval et al. (2018b) was the first to identify Uncertainty/Confusion about mental states as the MZ impairment via which CEA is associated to BPD. To further test this developmental model of MZ and clarify the associations between CEA, Uncertainty/Confusion about mental states in psychological difficulties, including BPD features, depression, and anxiety, the results of Duval et al. (2018b) need replication in a clinical sample of adolescents.

### The present study

The present study explored the relationships between CEA, MZ, BPD features, depression, and anxiety in adolescent mental health service users to examine whether MZ mediated the relationship between CEA and psychological difficulties. Given promising early findings that Uncertainty/Confusion mediated the relationship between CEA and psychological difficulties in a community sample of adolescents (Duval et al., 2018b), our first objective was to determine whether we could replicate these findings in a clinical sample of adolescents mental health service users. Our second objective was to examine whether Uncertainty/Confusion mediated the relationship between CEA and BPD features only or whether the RFQY Uncertainty/Confusion was a more general mediator of the relationships between CEA and psychopathology, including depression and anxiety symptoms in adolescents.

Based on previous literature, we hypothesized that (1) CEA would be associated with more borderline personality traits, depression, and anxiety symptoms as well as Uncertainty/Confusion and that (2) Uncertainty/Confusion would be associated with more borderline traits, depression, and anxiety, and that (3) Uncertainty/Confusion would mediate the relationship between CEA and psychological difficulties in a clinical sample of adolescents.

## Materials and methods

### Participants and procedure

We recruited adolescents (*N* = 94) aged 12–21 from an outpatient psychiatric clinic and a university psychology clinic in Quebec, Canada. Adolescents were informed of the ongoing research project and invited to participate. We obtained approval for the study from the Université Laval ethics committee. According to Article 21 of the province’s civil code, participants aged 14 and older have the right to consent. We obtained parental consent for participants aged 12–13. Adolescents completed a series of questionnaires via an electronic platform (Qualtrics). The only exclusion criteria were cognitive limitations which interfered with the ability to understand the questionnaires. In our final sample, the participants ranged in age from 12 to 21 years (*M* = 16.11, *SD* = 2.46). The sample was predominantly female (74%) and Caucasian French-Canadian (87%). In this sample, 61% of the participants had separated/divorced parents, and 36% were still living with both of their birth parents. Regarding treatment history, 83% of the participants had previously consulted mental health professionals.

### Measures

#### Mentalizing

The Reflective Function Questionnaire for Youth (RFQ-Y; Sharp et al., 2009; Duval et al., 2018a) is a self-report questionnaire designed to measure MZ in adolescents. The RFQ-Y comprises 25 items divided into three subscales reflecting Uncertainty/Confusion, interest/curiosity and certainty about mental states. The items are based on six-point Likert scales ranging from 1 (strongly disagree) to 6 (strongly agree). This questionnaire has been validated with clinical (Ha et al., 2013) and community (Duval et al., 2018a) samples showing adequate internal consistency ɑ = 0.89, ɑ = 0.75 and ɑ = 0.80. In this sample, the internal consistency of each subscale was, respectively, ɑ = 0.84, ɑ = 0.74 and ɑ = 0.72.

#### Emotional abuse

We assessed CEA using the “Childhood Experience of Care and Abuse Questionnaire” (CECA-Q; Bifulco et al., 2005). This questionnaire aims to examine the relationship between youth and their parental figures. CECA-Q has six subscales: antipathy, neglect, role reversal, psychological abuse, physical abuse, and sexual abuse. The frequency of each type of maltreatment is evaluated on a scale of four points (never, once, sometimes, often) about the mother and father. A total score is calculated for each type of abuse. This questionnaire has been validated with clinical (Smith et al., 2002) and community (Bifulco et al., 2005) samples. This study aggregated psychological abuse, antipathy, and neglect subscales into a composite CEA score. A similar procedure was previously used by Bottos and Nilsen (2014) and Duval et al. (2018b). In this sample, the internal consistency of the CEA score was ɑ = 0.74.

#### Borderline personality features

Borderline personality traits were measured using the French version of the Borderline Personality Features Scale for Children (BPFS-C; Crick et al., 2005; Ensink et al., 2020). The BPFS-C is a self-reported questionnaire assessing borderline personality traits in minors 9 years and older. This questionnaire is taken from the BPD subscale of the Personality Assessment Inventory (PAI; Morey, 2010). The BPFS-C has 24 items divided into four subscales of six items each. These scales are (1) affective instability, (2) identity issues, (3) negative relationships (4) self-harm. Each item uses a 5-point Likert scale ranging from 1 (not true at all) to 5 (always true). The total score is obtained by adding the four subscale scores. According to Chang et al., 2011, a total score of 66 represents the optimal cutoff score for discriminating BPD in adolescents. When validated by Ensink et al. (2020), the French version of the BPFS-C total score showed an internal consistency of ɑ = 0.91. In this sample, the total score of the BPFS-C was ɑ = 0.81.

#### Depression

We measured depression with the Depression subscale of the Beck Inventory for Youth (BYI; Beck et al., 2005). This widely used and well-validated self-report questionnaire measures depression symptoms, including youth’s negative emotions, thoughts and sleep disturbances. This questionnaire has 20 items and uses four-point Likert scales ranging from 0 (never) to 3 (always). The total score is obtained by adding the 20 response scores. These scores are transformed into T scores based on the gender and age of the participant. Scores of ≥ 70 are considered extremely elevated, 60–69 are moderately elevated, 55–59 are mildly elevated, and <55 are average. This questionnaire has excellent internal consistency (α = 0.95). In this sample, the BYI depression subscale showed an internal consistency of ɑ = 0.93.

#### Anxiety

We measured anxiety with the Anxiety subscale of the Beck Youth Inventory (BYI; Beck et al., 2005). This self-report questionnaire measures physiological symptoms of anxiety, worries and fears regarding the future, loss of control, peers’ reactions and school performance. Like the depression inventory, this questionnaire has 20 items, uses four-point Likert scales ranging from 0 (never) to 3 (always) and follows the same interpretation guidelines. The BYI anxiety subscale in this sample showed excellent internal consistency ɑ = 0.91.

### Data analysis

Descriptive statistics, correlations and assumptions were examined in SPSS 28 (IBM). We tested a pathway from CEA to pathology via MZ using a path analysis model with a maximum likelihood estimation method in Mplus 8.5 (Muthén and Muthén, 1998). The model tested indirect effects (which involve the same calculations as mediation analysis) from the predictor (CEA) to the outcomes (borderline traits, depression, anxiety) through adolescent MZ impairment scales (Uncertainty/Confusion) as a potential mediator. All indirect effects were bootstrapped 1,000 times with 95% confidence intervals (CIs). These indirect effects were considered significant if the 95% confidence intervals did not include zero (Fairchild and MacKinnon, 2009). In order to test the fit of the model, we used the Comparative Fit Index (CFI; ≥ 0.90), the Tucker-Lewis Index (TLI; ≥ 0.90), the Root Mean Square Error of Approximation (RMSEA; ≤ 0.06), the Standardized Root Mean Square (SRMR; ≤ 0.08), the chi-square (χ2) goodness of fit index and the chi-square to degrees of freedom ration (χ2/df) following guidelines of Kline (2016).

## Results

### Relationships between emotional abuse, mentalizing and psychopathology

Correlations between the key study variables are presented in Table 1. Uncertainty/Confusion was, as expected, positively correlated with borderline traits, depression, anxiety and CEA. CEA was positively correlated with BPD features, depression and anxiety. Finally, as age was moderately correlated with Uncertainty/Confusion about mental states and CEA, these relationships were controlled for in the further analysis.

**Table 1:** Descriptive statistics and correlations for key study variables.

	*M*	*SD*	1.	2.	3.	4.	5.	6.	7.	8.
1. Age	16.13	2.46	—							
2. RFQY-U/C	3.80	0.91	−0.20	—						
3. RFQY-I/C	4.54	0.74	0.19	−0.21^*^	—					
4. RFQY-Certainty	3.39	0.83	0.12	−0.13	0.10	—				
5. BPD features	64.80	13.75	0.04	0.58^***^	−0.11	−0.19	—			
6. Depression	62.93	11.74	−0.10	0.44^**^	−0.11	−0.18	0.52^**^	—		
7. Anxiety	61.42	12.69	−0.06	0.42^**^	0.20	−0.16	0.47^**^	0.73^**^	—	
8. CEA	73.81	30.02	0.20	0.25^*^	0.11	0.06	0.37^**^	0.16	0.32^*^	—

RFQY, Reflective Function Questionnaire for Youth; U/C, RFQY Uncertainty/Confusion scale; I/C, RFQY Interest/curiosity scale; BPD features, borderline personality features; CEA, Childhood Emotional Abuse; M, Mean; SD, Standard Deviations. ^**^p < 0.01; ^*^p < 0.05.

### Path analysis

We conducted a path analysis to test whether Uncertainty/Confusion mediates the relationships between CEA and borderline traits, depression and anxiety in adolescence. All fit indices showed an excellent model fit, χ^2^(3) = 0.633, *p* = 0.89, χ^2^/df = 0.211, CFI = 1.00, TLI = 1.00, RMSEA < 0.001, SRMR = 0.013. Furthermore, after examining covariances between outcome variables, significant relationships were observed between BPD features and depression (*β* = 0.354; *p* < 0.001), BPD features and anxiety (*β* = 0.263; *p* = 0.012) and depression and anxiety (*β* = 0.673*; p* < 0.001).

**Figure 1:**
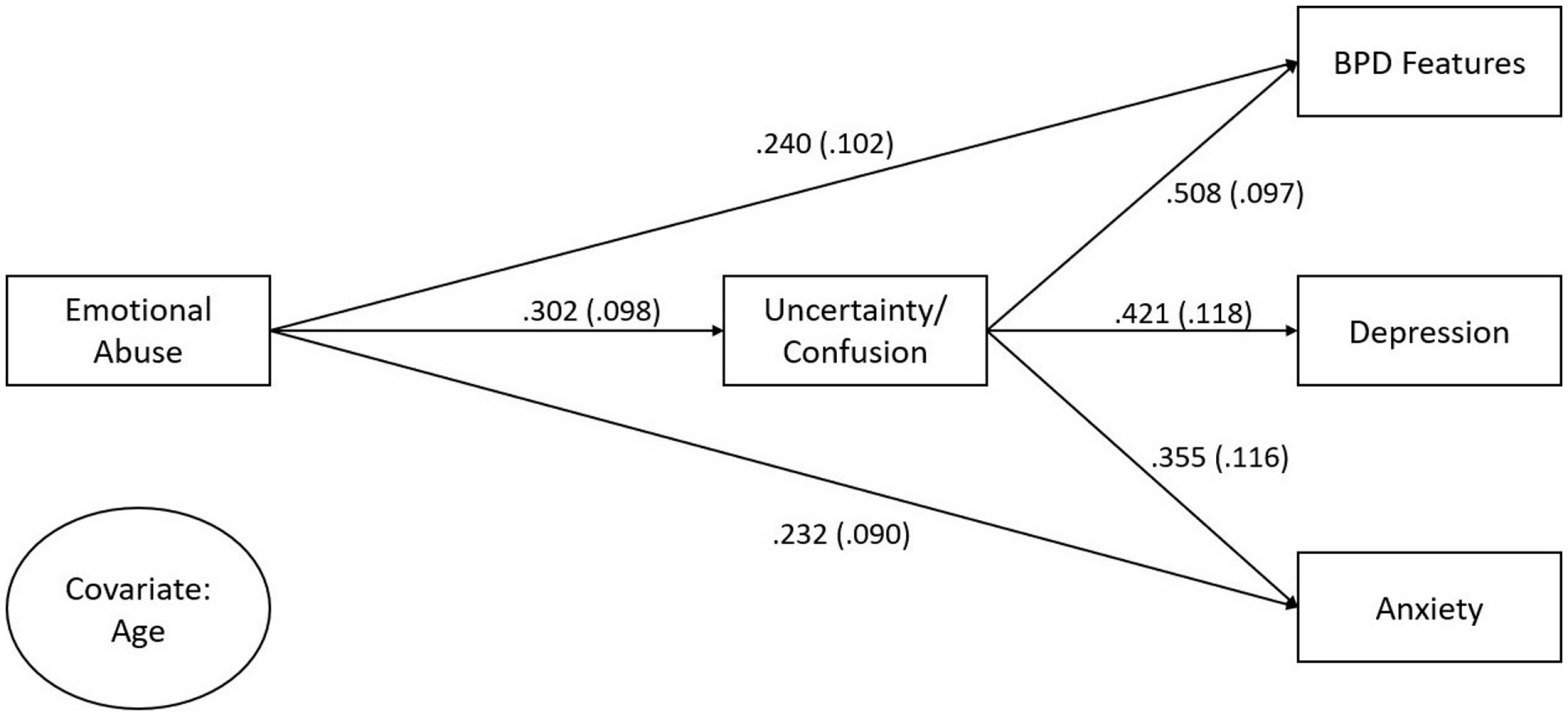
Path analysis from CEA to adolescent borderline personality features, depression and anxiety with mentalizing (uncertainty/confusion) as a mediator. Covariate age predicted uncertainty/confusion and emotional abuse. Parameters are standardized.

Figure 1 shows the results of the path analysis. Results show that CEA had a direct effect on Uncertainty/Confusion (*β* = 0.302; *p* = 0.002), BPD features (*β* = 0.240*; p* = 0.018) and anxiety (*β* = 0.232; *p* = 0.010). Moreover, Uncertainty/Confusion had a direct effect on BPD features (*β* = 0.508; *p* < 0.001). CEA also had a significant indirect effect on BPD features via Uncertainty/Confusion (*b* = 0.070, 95% CI [0.021, 0.131]), accounting for 39% of the total effect. The total effect of CEA on adolescent BPD features was reduced from *β* = 0.394 to a direct effect *of β* = 0.240. The model explained 38% of the variance of adolescent borderline personality traits.

With regard to depression, Uncertainty/Confusion had a significant direct effect (*β* = 0.421, *p* < 0.001). However, the direct effect of CEA on depression was not significant (*β* = 0.064; *p* = 0.489). CEA had a significant indirect effect on depression through Uncertainty/Confusion (*b* = 0.050, 95% CI [0.012, 0.107]). This indirect effect accounted for 66% of the total effect of CEA on depression symptoms. The total effect of CEA on depression was reduced from β = 0.191 to a direct effect of *β* = 0.064. The model explained 20% of depression symptoms variance.

Regarding anxiety, Uncertainty/Confusion had a significant direct effect on anxiety symptoms (*β* = 0.355, *p* = 0.002). The indirect effect of CEA on anxiety through Uncertainty/Confusion was also significant (*b* = 0.045, 95% CI [0.009, 0.103]). The indirect effect accounted for 32% of the total effect of CEA on anxiety symptoms. The total effect of CEA on anxiety was reduced from *β* = 0.339 to a direct effect of *β* = 0.232. The model explained 22% of the variance in anxiety symptoms.

## Discussion

This study examined the relationships between CEA, MZ, BPD features, depression and anxiety symptoms. The key study findings are that in adolescent mental health service users, Uncertainty/Confusion about mental states partially mediated the relationship between CEA and BPD features and the relationships between CEA and anxiety. In addition, there was an indirect effect where CEA predicted Uncertainty/Confusion, which then predicted depressive symptoms.

Regarding the relationships between CEA, MZ and psychopathology, as hypothesized, CEA was associated with more Uncertainty/Confusion, as well as with more BPD features and anxiety symptoms. The findings are consistent with previous research showing that CEA is associated with general MZ difficulties (Quek et al., 2017; Schwarzer et al., 2021). The current study adds to previous research by showing that CEA induces MZ difficulties characterized by Uncertainty/Confusion about mental states and replicates the findings of Duval et al. (2018b), who found associations between CEA and Uncertainty/Confusion in a community sample. The association between CEA and psychopathology found in the present study extends previous findings that CEA is a risk factor for BPD features (Bounoua et al., 2015; Duval et al., 2018b; Porter et al., 2020) and anxiety (Banducci et al., 2017; Guo et al., 2021).

Turning to the relationships between MZ and psychopathology, as hypothesized, there was a positive association between Uncertainty/Confusion and BPD features, depression and anxiety. This replicates and extends previous research showing associations between Uncertainty/Confusion about mental states, adolescent BPD features, and Internalizing difficulties (Duval et al., 2018a,b). The present study presents new evidence showing an association between Uncertainty/Confusion about mental states and depression and anxiety symptoms. These findings broadly align with previous research showing associations between general MZ deficits and depression (Belvederi Murri et al., 2016) and anxiety (Ballespi et al., 2018; Seyed Mousavi et al., 2021). However, our findings indicate that Uncertainty/Confusion are important MZ difficulties associated with depression and anxiety.

A key finding of the study was that CEA and Uncertainty/Confusion about mental states explained 38% of the variance in BPD features. This finding is consistent with the MZ model of BPD (Fonagy and Luyten, 2009; Bateman and Fonagy, 2016), according to which early adverse experiences undermine the development of full MZ capacities, thereby increasing vulnerability to BPD symptoms. Uncertainty/Confusion about mental states partially mediated the relationship between CEA and BPD features. This aligns with previous research showing a mediating effect of Uncertainty/Confusion between CEA and BPD features in community adolescents (Duval et al., 2018b) and a mediating effect of general MZ deficits in clinical adolescents (Quek et al., 2017). The findings from the current study show that Uncertainty/Confusion is strongly associated with BPD features in a clinical sample and replicates that of Duval et al. (2018a) in a community sample. While many studies on adolescent BPD focus on hypermentalizing (McLaren et al., 2022), our findings suggest that Uncertainty/Confusion about mental states may also be a crucial mentalizing difficulty associated with BPD in adolescents.

Regarding anxiety, Uncertainty/Confusion of mental states was observed to partially mediate the relationship between CEA and anxiety symptom severity. Indeed, CEA and Uncertainty/Confusion of mental states explained 22% of the variance in anxiety symptoms. This is a new finding since, to our knowledge, no other study has tested such a model. Our findings showing an association between poor MZ and anxiety in adolescents extends previous research (Ballespi et al., 2018; Seyed Mousavi et al., 2021) by showing that Uncertainty/Confusion about mental states is the MZ difficulty associated with anxiety in adolescents. One way of understanding these findings is that CEA, where attachment figures misattribute hostile intentions and qualities to a child or youth, negatively impacts MZ by creating confusion about their own minds and that of others. This, in turn, then contributes to hypervigilance and anxiety symptoms.

Regarding depression, CEA predicted Uncertainty/Confusion about mental states and Uncertainty/Confusion was associated with depressive symptoms. CEA and Uncertainty/Confusion about mental states explained 19% of the variance in depression symptoms. This finding builds on previous research showing associations between childhood trauma, poor general MZ capacities and depression symptoms in a clinical sample of adolescents (Belvederi Murri et al., 2016). It is consistent with previous findings that mentalizing regarding others mediated the relationship between sexual abuse and depressive symptoms in school-aged children (Ensink et al., 2015). While we did not find mediation, the study adds new knowledge by showing that in adolescents consulting mental health services, a specific type of mentalizing difficulty, namely Uncertainty/Confusion about mental states, is associated with CEA and depressive symptoms. In the present study, the association between Uncertainty/Confusion and depressive symptoms was strong, suggesting that mentalizing difficulties characterized by Uncertainty/Confusion are implicated in depressive symptoms.

Together, the findings suggest that Uncertainty/Confusion about mental states is associated with CEA and that Uncertainty/Confusion is a transdiagnostic risk factor associated with a range of mental health difficulties, including BPD features, depression and anxiety in adolescents. The study findings have implications for clinical interventions with adolescents who have experienced CEA and present with BPD features, depression, and anxiety symptoms. If we consider that CEA undermines one’s confidence in knowing who you are, what you feel and what your intentions are, interventions that nurture trust in their sense of self and MZ capacities may be crucial to help adolescents recover MZ. Interventions to reduce Uncertainty/Confusion about self and others by scaffolding trust in their MZ abilities may reduce anxiety and depressive symptoms, as well as emotional dysregulation. Furthermore, helping adolescents mentalize regarding CEA and its impact may also facilitate understanding and contribute to recovery. Mentalization Based-Treatment (MBT) might be particularly useful in developing more robust, agentful MZ capacities, thus reducing Uncertainty/Confusion in adolescent patients.

Although this study has several strengths, including the use of a difficult-to-recruit sample of adolescents consulting with psychiatric and psychology services, some limitations must be considered before generalizing the study findings. The study’s cross-sectional nature limits conclusions regarding the directionality of the links made. While our findings are consistent with mediation, a longitudinal research design is required to confirm mediation and establish causality between these variables. Second, while the sample size was adequate to test the model, the findings need to be replicated with a larger sample size as we lacked the statistical power to detect significance when associations were weak to medium. In addition, our sample primarily consisted of female participants (74%), limiting the extent to which the findings can be generalized to males. Further research is needed to examine gender effects and whether mediation is associated with gender differences in adolescents’ MZ capacities and psychopathology. In addition, while this study focused on the RFQY Uncertainty/Confusion subscale, further research should examine the Interest/Curiosity and Certainty about mental states subscales and their relationships with psychopathology. Finally, the construct of Uncertainty/Confusion about mental states needs further clarification.

## Conclusion

This study provides new evidence that Uncertainty/Confusion about mental states in the context of CEA is a transdiagnostic risk factor for the emergence of mental health problems in adolescents, including BPD features, depression and anxiety. CEA, when parents mentally attack and misattribute their hostile intentions to the child or adolescent, induces confusion and damages the development of confidence in knowing who they are, what they feel and what their own intentions and that of others are. The findings have important clinical implications and suggest that interventions that target the clinical outfall of CEA and scaffold the development of mentalizing about self and others could reduce psychological distress and dysfunction in youth.

## Data availability statement

The original contributions presented in the study are included in the article/supplementary material, further inquiries can be directed to the corresponding author.

## Ethics statement

The studies involving human participants were reviewed and approved by Laval University Ethics Committee. Written informed consent to participate in this study was provided by the participants’ legal guardian/next of kin.

## Author contributions

All authors listed have made a substantial, direct, and intellectual contribution to the work and approved it for publication.

## Conflict of interest

The authors declare that the research was conducted in the absence of any commercial or financial relationships that could be construed as a potential conflict of interest.

## Publisher’s note

All claims expressed in this article are solely those of the authors and do not necessarily represent those of their affiliated organizations, or those of the publisher, the editors and the reviewers. Any product that may be evaluated in this article, or claim that may be made by its manufacturer, is not guaranteed or endorsed by the publisher.
